# Attitude and Usage of Mobile Phone among Students in Yazd University of Medical Science

**DOI:** 10.5812/ircmj.4141

**Published:** 2013-08-05

**Authors:** Mohamad Hosein Baghianimoghadam, Hasan Shahbazi, Dariush Masoodi Boroojeni, Behnam Baghianimoghadam

**Affiliations:** 1 Faculty of Health, Shahid Sadoughi University of Medical Sciences and Health Services, Yazd, IR Iran; 2 Reaserch Consultant, Shahid Sadoughi University of Medical Sciences and Health Services, Yazd, IR Iran

**Keywords:** Cellular Phone, Attitude, Universities, Students

Dear Editor

Mobile phones as a symbol of electronic century and digital world has spared worldwide especially in youth (1, 2). Subscribers of mobile phones were increased from 12.4 million in 1990 to 500 million in 2000 to 3.3 billion in 2008 and 5.3 billion at the end of 2010 (3-6). Estimations show that the prevalence of mobile use will be increased to 95% till 2013 (2). In Iran Subscribers were increased from 4.3 million in 2004 to 41 million in 2008; it is estimated that about 53 – 55 million are subscribers of mobile phone in Iran now (2). Communication technologies (that causes to higher level in quality of life) have several benefits in our modern life; but we can’t disregard some negative effects of mobile phones that caused serious social and cultural problems in communities (2). This study tries to detect the attitude and practices among students of Yazd University of medical sciences were living in dormitories about using mobile phones.

This cross sectional study was done on 245 students, were living in university dormitories of Shahid Sadoughi University of Medical Sciences. A researcher designed a questionnaire to collect data. Internal validity of the questionnaire was confirmed by a team of experts in communication science and health education. Its external validity also confirmed by a pilot study, using a group of students whom were not participating in the main study (Alpha chornbach = 0.72). The questionnaire was consisted in three parts: demographic data, 11 questions on attitude and 10 questions about practice. All registered data were analyzed by ANOVA (for analysis of replies to attitude questions according to sex) and Chi-square (analysis of GPA difference) tests under SPSS software. Significant level was considered as P < 0.05.

Mean age of participants was 21.91 ± 3.01. From all 245 participants in study, 155 (63.3%) were girls and 90 (36.7%) were boys; 94 (38.4%) from Faculty of Health, 44 (18%) from Faculty of Nursing & Midwifery, 62 (25.3%) from Faculty of Para medicine Sciences, 24 (9.8%) from Faculty of Medicine and 15 (6.1%) from Faculty of Dentistry. 6 participants (2.4%) didn’t mention their faculty. 26 (10.6%) were student in associate degree, 176 (71.8%) in BS (Bachelor of Science), 6 (2.4%) in MSc (Master of Science) and 37 (15.1%) were student of medicine and dentistry. 211 participants (86.1%) were single and 31 (12.7%) were married. GPA (Grade Points Average) of participants in previous semester were lower than 16 (from 20) in 42 (17.4%), between 16-18 in 101 (41.2%), and higher than 18 in 45 (18.37%) participants. 57 participants (23.29%) had not mentioned their GPA in questionnaire. Mean GPA in women was higher than men (P = 0.005).

195 participants’ families (79.6%) were living in cities and 46 (18.8%) in villages. 18 (7.3%) assessed their family economic status as affluent, 58 (23.7%) as relatively affluent, 149 (60.8%) as average income and 16 (6.5%) as low income. 240 (98%) of all participants had mobile phones and only 5 (2%) were not mobile subscribers. Mean scores of attitude in men and women were 41.82 ± 4.3 and 40 ± 4.4 (P = 0.003); also mean scores for practice were 6 ± 4 and 7.7 ± 3.8 respectively (P = 0.002). Based on our study about 97% of students have mobile phones and there were no significant differences between sexes in this context. Based on our study and some other studies there were significant differences between sex and attitude and also sex and practice (7). In our study more than 87% of participant said that mobile phone is a necessary instrument for students while only 5% think that it is not necessary; this rate in other some other studies was similar to ours (2).

In Shamszadeh et al. (8) study about the role of information technology on quality of life of families mobile phone and SMS are as cause of destroying traditional form of family. In our study about 59% of students accepted that mobile phone is a cause of inappropriate relationship between boys and girls. More than 87% of participants believed that mobile phone is not an appropriate place to store information; in another study 28% were agreed and 72% disagreed this question (9).

While about 17% of our participants believed that life without mobile phone is more comfortable, 62% didn’t have such a belief. These results are similar to some other studies (1). More than 23% of participants expressed that mobile phone have severe adverse effects on their study and academic achievement while 54% rejected such influence. About 19% of our participants had tried to decrease use of mobile phone but were unsuccessful; this rate in Thomee study (10) was 10%. Based on our results, students must become familiar with advantages and disadvantages of mobile phones. This must be done by media, mobile phone companies and educational packages with sim cards by telecommunication service organizations. This can help to improve the culture of using new technological instruments and then community health promotion.[Table tbl6830]

**Table 1. tbl6830:** Rate of Answers According to Sex and Their Related P Values

Question	Strongly agree		Agree		No comment		Disagree		Strongly disagree		P value
	girls	Boys	girls	boys	girls	boys	girls	boys	girls	boys	
**Mobile phone is necessary for each university student, No. (%)**	76 (50)	43 (51.2)	61 (40.1)	26 (31)	8 (5.3)	10 (11.9)	6 (3.9)	5 (6)	1 (0.7)	0 (0)	0.249
**Mobile phone is one of main causes of inappropriate relationship between boys and girls, No. (%)**	41 (27)	19 (22.6)	56 (36.8)	23 (27.4)	20 (13.2)	12 (14.3)	25 (16.4)	10 (11.9)	10 (6.6)	20 (23.8)	0.004
**Using mobile phone must have age limitation, No. (%)**	63 (41.4)	24 (28.6)	59 (38.8)	28 (33.3)	18 (11.8)	15 (17.9)	10 (6.6)	7 (8.3)	2 (1.3)	10 (11.9)	0.002
**Mobile phone is not appropriate place for confidential information to store, No. (%)**	94 (61.8)	47 (56)	46 (30.3)	19 (22.6)	7 (4.6)	14 (16.7)	3 (2)	3 (3.6)	2 (1.3)	1 (1.2)	0.028
**Mobile phone can be addictive, No. (%)**	65 (42.8)	30 (35.7)	60 (39.5)	36 (42.9)	14 (9.2)	5 (6)	9 (5.9)	5 (6)	4 (2.6)	8 (9.5)	0.159
**Life without mobile phone is better, No. (%)**	8 (8.6)	13 (9.5)	4 (9.9)	15 (4.8)	33 (21.7)	16 (19)	58 (38.2)	24 (29.6)	33 (21.7)	32 (38.1)	0.069
**Mobile phone can have serious damages except its benefits, No. (%)**	63 (41.4)	25 (29.8)	65 (42.8)	37 (44)	14 (9.2)	16 (19)	9 (5.9)	3 (3.6)	1 (0.7)	3 (3.6)	0.051
**Mobile phone have less problematic effects on boys than girls, No. (%)**	13 (8.6)	12 (14.3)	27 (17.8)	15 (17.9)	45 (29.6)	38 (45.2)	43 (28.3)	11 (13.1)	24 (15.8)	8 (9.5)	0.014
**I fell anxious and guilty because of what I do with my mobile phone, No. (%)**	11 (7.2)	4 (4.8)	8 (5.3)	7 (8.4)	21 (13.8)	13 (15.7)	59 (38.8)	32 (38.6)	53 (34.9)	27 (32.5)	0.818
**When someone calls me I feel self-esteem, No. (%)**	25 (16.4)	12 (14.5)	45 (29.6)	29 (34.9)	39 (25.7)	21 (25.3)	23 (15.1)	10 (12)	20 (13.2)	11 (13.3)	0.912
**If for long time anyone not call or not send SMS to me, I will feel depressed, No. (%)**	24 (15.8)	12 (14.5)	43 (28.3)	20 (24.1)	28 (18.4)	18 (21.7)	26 (17.1)	23 (27.7)	31 (20.4)	10 (12)	0.219
